# Correlates of climate change distress: The difference to general distress

**DOI:** 10.1016/j.ijchp.2025.100613

**Published:** 2025-08-05

**Authors:** Jil Beckord, Nadja Gebhardt, Christoph Nikendei, Julia Barbara Krakowczyk, Eva-Maria Skoda, Martin Teufel, Alexander Bäuerle

**Affiliations:** a Clinic for Psychosomatic Medicine and Psychotherapy, LVR-University Hospital Essen, University of Duisburg-Essen, Essen, Germany; b Center for Translational Neuro- and Behavioral Sciences (C-TNBS), University of Duisburg-Essen, Essen, Germany; c Department for General Internal Medicine and Psychosomatics, University Hospital Heidelberg, Heidelberg, Germany

**Keywords:** Climate crisis, Mental health, Protective factors, Risk factors, Eco-anxiety

## Abstract

**Introduction:**

Climate change has significant consequences on mental health, which are summarized under concepts like eco-anxiety or climate change distress. However, these recently developed concepts still suffer from a lack of clarity.

**Aim:**

The aim of this study is to improve the conceptual clarity of climate change distress through analysing its’ correlations with various psychological and demographic factors. In this context, the specific associations of climate change distress are compared to those of general distress.

**Methods:**

In a cross-sectional study *N* = 1000 participants completed an online questionnaire. Climate change distress was assessed using the ‘Climate Change - Man-Made Disaster-Related Distress Scale’. General distress was assessed using the Distress Thermometer, the Generalized Anxiety Disorder scale, and the Patient Health Questionnaire. Several measurement instruments were examined as possible correlates. The outcomes were investigated using multiple linear regression models.

**Results:**

Relevant correlates of climate change distress included trust in government to handle climate change and several emotion regulation strategies. The associated factors of general distress were distinct from those of climate change distress, such as gender and sense of coherence.

**Discussion:**

The results suggest that the correlates of climate change distress differ from those of general distress. This implies that climate change distress and general distress are two related, however distinct constructs. The associated factors can be promising targets for psychotherapy and intervention strategies.

## Introduction

Climate change is viewed as the global risk with the most significant consequences in the next decade (World Economic Forum, 2023) and poses a fundamental threat to human health (World Health Organization, 2023). There are vast physical and mental health consequences which are in an interconnected relationship (Clayton et al., 2021; Watts et al., 2015; World Health Organization, 2023). Regarding mental health, climate change has both direct and indirect negative consequences (Cianconi et al., 2020; Gebhardt et al., 2023; World Health Organization, 2023). The mental health consequences resulting from the direct experience of extreme weather events and climate catastrophes include post-traumatic stress disorder, anxiety, and depression disorders (Clayton, 2021; Gebhardt et al., 2023; Lawrance et al., 2022). The consumption of information about climate change and the awareness of negative present and future consequences can also lead to indirect detrimental consequences on mental health, including worries, anxiety, uncertainties, or even pre-traumatic stress (Bunz, 2016; Clayton, 2020; Schwaab et al., 2022). The acknowledgement of climate change as a fundamental threat, while at the same time experiencing a lack of environmental self-efficacy, can trigger distressing emotional reactions and worries about the future, including help- and hopelessness and a sense of impending doom (Clayton et al., 2017; Clayton et al., 2021; Sawitri et al., 2015).

The different emotions associated with climate change are recognized and summarized under new terms like “eco-anxiety”, “eco-anger”, “climate distress”, or “solastalgia” (Albrecht, 2005, 2011; Albrecht et al., 2007; Clayton et al., 2017; Clayton et al., 2021; Pihkala, 2022; Searle & Gow, 2010). While climate change distress and eco-anxiety gain relevance in research and health care and are sometimes even used as umbrella terms for negative emotions associated with climate change (Koder et al., 2023), they still suffer from a lack of clarity regarding their concepts and specific predictors (Boluda-Verdu et al., 2022; Coffey et al., 2021). Previous attempts to clarify these terms focused on eco-anxiety and provided an overview of its’ definitions, characteristics, and associations in literature (Boluda-Verdu et al., 2022; Coffey et al., 2021). However, an attempt to specify climate change distress (in the following “CC distress”; (Boluda-Verdu et al., 2022)) and investigate its’ relevant associated factors is still missing.

Based on associated emotions, there should be a clear distinction of CC distress not only towards eco-anxiety, but also towards related general constructs such as general distress (Boluda-Verdu et al., 2022). One study investigated such distinctions between climate emotions and general emotions and showed that, even though they seem to correspond in their experience, they are associated with different behaviours (Contreras et al., 2024). In an earlier study, low correlation indices between the CC distress measure ‘Climate Change – Man-Made Disaster-Related Distress Scale’ (CC-MMDS) and measures of general distress suggested that the two concepts overlap but are distinct, as well (Beckord et al., 2024). With climate change consequences on the rise, more and more people will experience CC distress. However, psychotherapists still often feel inadequately prepared to address these issues in psychotherapeutic contexts (Trost et al., 2024). It is, therefore, essential to understand the specific factors that promote CC distress or that have the possibility to protect from it. To investigate associated factors of CC distress and to improve targeted psychotherapy and interventions, more research is needed.

Therefore, the aim of the present study is to differentiate CC distress from general distress and to investigate specific associated factors. In order to do this, associated factors are compared between the concepts CC distress and general distress through multiple regression analyses. General distress is operationalized through measures of distress, anxiety, and depression to have a more thorough overview and a more specific distinction.

## Methods

This study received approval from the Ethical Committee of the Medical Faculty of the University of Duisburg-Essen (23–11,342-BO), has been carried out in accordance with the Declaration of Helsinki, and is reported according to the ‘Strengthening the Reporting of Observational Studies in Epidemiology’ (STROBE) guidelines for cross-sectional studies (Von Elm et al., 2007) (supplementary material). The analyses and results presented in this paper are a follow-up to a primary paper that used part of the data on the validation of the CC-MMDS (Beckord et al., 2024).

### Participants and procedure

The study was distributed via the digital survey platform Unipark (Tivian XI GmbH, 2023). Recruitment took place from October 2023 to October 2024 across various public and work-related channels. Participants had to be at least 18 years old, possess an Internet-capable device, and proficient command of the German language to be eligible. Participation was anonymous and voluntary. All participants gave their electronic informed consent. Out of 1328 participants initially engaged, 1000 successfully completed the survey, reflecting a completion rate of 75.3 %. Another 17 participants were excluded from further analysis due to a reported age under 18. Additionally, data was scrutinized for indications of careless and inattentive responding, specifically by using the a priori index of instructed response items and the post-hoc index of invariability according to Ward and Meade (2023). Invariability was detected by computing a longstring index (Ward & Meade, 2023). Participants were excluded if they had a string responding of > 16, resulting in the exclusion of 42 participants. Participants were also excluded if they answered either of two instructed response items wrong (“Please choose “I totally agree” to show that you pay attention.”/”Please choose “I totally disagree” to show that you pay attention.”), leading to the exclusion of an additional 37 participants. This resulted in a remaining sample size of *N* = 904 (90.4 %) participants. The sample size was sufficient for the planned analyses as according to Green’s rule of thumb a number of *N* > 194 is sufficient for detecting a medium effect with 18 predictors (50 + 8 × 18 = 194) and even with a preferred 20:1 sample-to-variable ratio the sample size is *N* > 360 (Memon et al., 2020).

### Measures

Sociodemographic variables, such as gender, age, educational level, marital and occupational status, and existence of a mental health disorder were assessed. Participants who indicated that they had been diagnosed with a mental disorder were asked to specify the diagnosis in a free-text field. These free-text responses were categorized into broader diagnostic groups for descriptive purposes. Additionally, participants were asked to indicate how they would position themselves with regard to climate change (I see myself “strongly as a climate change denier” / “more as a climate change denier” / “neutral/neither” / “more as a climate change activist” / “strongly as a climate change activist”), and how much time they spend each week on informing themselves about climate change. No additional definitions of “climate change denier” or “climate change activist” were provided to the participants, as the intention was to capture participants’ intuitive understanding and subjective identification with these socially salient roles. To assess the outcomes CC distress and general distress, the following validated instruments were applied.

*Climate Change – Man-Made Disaster-Related Distress Scale* (CC-MMDS) (Beckord et al., 2024). The CC-MMDS is a German validated instrument measuring CC distress on two subscales: psychological distress (CC-MMDS-PD, 11 items) and change of existing belief systems (CC-MMDS-CBS, 5 items). The 16 items are answered on a 7-point Likert scale ranging from 1 = “does not apply at all” to 7 = “applies fully”. The internal consistencies in this study were α = .94 for the total scale, α = .93 for the CC-MMDS-PD subscale, and α = .87 for the CC-MMDS-CBS subscale.

*Generalized Anxiety Disorder Scale* (GAD-7) (Spitzer et al., 2006). The GAD-7 is an established validated instrument that assesses anxiety symptoms with seven items. The items can be answered on a 4-point Likert scale ranging from 0 = “never” to 3 = “nearly every day”. Cut-off sum scores indicate mild ≥ 5, moderate ≥ 10, and severe ≥ 15 generalized anxiety. The internal consistency in the present study was α = .89.

*Patient Health Questionnaire-8* (PHQ-8) (Kroenke et al., 2009). The PHQ-8 is an established validated screening instrument to assess depression symptoms. It consists of eight items that can be answered on a 4-point Likert scale ranging from 0 = “never” to 3 = “nearly every day”. Cut-off sum scores indicate mild ≥ 5, moderate ≥ 10, moderately severe ≥ 15, and severe ≥ 20 depression. The internal consistency in the present study was α = .88.

*Distress Thermometer* (DT) (Mehnert et al., 2006). The DT is an established validated instrument that measures psychological distress on one visual scale ranging from 0 = “no distress” to 10 = “extreme distress”.

The following instruments were investigated as possible associated factors.

*Trust in government* (Bäuerle et al., 2020). This scale assesses trust in governmental actions to face COVID-19 via four items. In the present study, the scale was adapted to climate change. The items are answered on a 7-point Likert-scale ranging from 1 = “complete disagreement” to 7 = “complete agreement”. The internal consistency in this study was α = 0.67.

*Subjective level of information* (Bäuerle et al., 2020). This scale assesses the subjective level of information regarding COVID-19 via three items. In the present study, the scale was adapted to climate change. The items are answered on a 7-point Likert-scale ranging from 1 = “complete disagreement” to 7 = “complete agreement”. The internal consistency in this study was α = 0.79.

*Cognitive Emotion Regulation Questionnaire* (CERQ) (Garnefski et al., 2001). The CERQ assesses nine dimensions of cognitive emotion regulation and was used in this study with the short form (Garnefski & Kraaij, 2006a) of the German adaptation (Loch et al., 2011). In the present study, the scale was adapted to climate change. The 18 items are answered on a 5-point Likert-scale from 1 = “(almost) never” to 5 = “(almost) always”. The internal consistencies in this study were α = 0.77 for the total scale, r_SB_^1^ = 0.70 for acceptance, r_SB_ = 0.67 for rumination, r_SB_ = 0.60 for positive reappraisal, r_SB_ = 0.63 for self-blame, r_SB_ = 0.78 for positive refocusing, r_SB_ = 0.81 for catastrophizing, r_SB_ = 0.75 for blaming others, r_SB_ = .77 for refocus on planning, and r_SB_ = 0.74 for putting into perspective.

*Sense of Coherence Scale – Short Form* (SOC-L9) (Schumacher et al., 2000). The unidimensional SOC-L9 measures sense of coherence (SOC) and is a revised short form of the sense of coherence scale by Antonovsky (1987). It consists of nine items that can be answered on a 7-point Likert scale with different answer formats. The internal consistency in this study was α = .86.

*Locus of Control* (LOC) (Rotter, 1966). The LOC is a validated instrument to assess LOC via the two factors internal and external LOC. In this study the German short form “*Kurzskala Interne und Externe Kontrollüberzeugungen*” was used (Jakoby & Jacob, 2002). The six items are answered on a 5-point Likert-scale ranging from 1 = “totally agree” to 5 = “totally disagree”. The internal consistencies in this study were α = 0.63 for the total scale, α = 0.63 for internal LOC, and α = 0.48 for external LOC.

*Resilience Scale* (RS-11) (Schumacher et al., 2005). The RS-11 is the unidimensional German validation of the original instrument by Wagnild and Young (1993). The RS-11 measures resilience with eleven items that can be answered on a 7-point Likert-scale ranging from 1 = “disagree” to 7 = “agree”. The internal consistency in this study was α = 0.87.

*General Self-Efficacy Scale* (GSE) (Schwarzer & Jerusalem, 1999). The GSE measures general self-efficacy with ten items. The items can be answered on a 4-point Likert-scale ranging from 1 = “not at all true” to 4 = “exactly true”. The internal consistency in the present study was α = 0.87.

### Data analysis

Data analysis was carried out using R version 4.2.2 (R Core Team, 2022). Only complete data sets were used for analysis and mean values and percentages were used to describe the sociodemographic characteristics. To differentiate the correlates of CC distress from those of general distress, four linear regression models were calculated to investigate individual contributions of associated factors to CC-MMDS, as measure for CC distress, and DT, GAD-7, and PHQ-8, as measures for general distress. For exploratory reasons, additional regression models with each of the subscales of the CC-MMDS were calculated. For detailed results, see supplementary material. As possible associated factors, age and gender, as well as the questionnaires trust in government, subjective level of information, CERQ, SOC-L9, LOC, RS-11, and GSE were used. The gender category inter/diverse was excluded for the regression analyses as the sample size in this category was too small for a meaningful comparison (*n* = 19). In order to enable comparability of coefficients, regression variables were standardized. It was checked whether the requirements for a regression were fulfilled. The Breusch-Pagan-Test was significant and revealed that homoscedasticity was not fulfilled. Therefore, robust standard errors were calculated. There was no autocorrelation as the Durbon-Watson statistic was approximately 2 and not significant. There was also no multi-collinearity as variance inflation factors < 10. Residuals were approximately normally distributed and no influencing cases were excluded as there was no indication of implausibility. To control for Type I error due to multiple testing, all 108 *p*-values (18 predictors across 6 regression models) were adjusted using the Bonferroni-Holm correction.

## Results

### Sample characteristics

The final study sample (*N* = 904) had a mean age of *M* = 39.75 (*SD* = 15.7) with a range between 18 and 93 years. 615 (68.03 %) participants identified as female, 270 (29.87 %) as male, and 19 (2.1 %) as inter/diverse. Additional characteristics are displayed in Table 1. Next to sociodemographic characteristics, specific information regarding the topic climate change was assessed. The study sample had a mean of *M* = 4.30 (*SD* = 1.11) regarding the whole scale of the CC-MMDS, *M* = 3.99 (*SD* = 1.18) regarding the subscale CC-MMDS-PD, and *M* = 5.00 (*SD* = 1.18) regarding the subscale CC-MMDS-CBS. Regarding the general distress measures, the study sample had a mean sum score of *M* = 6.38 (*SD* = 3.84) in GAD-7, *M* = 6.10 (*SD* = 3.89) in PHQ-8, and a mean score of *M* = 5.21 (*SD* = 2.14) in DT.

**Table 1:** Demographic characteristics of the study sample (*N* = 904).

Categorical variables	*n* ( %)
Educational level	
University degree	560 (61.95)
University entrance qualification	260 (28.76)
Upper secondary school	52 (5.75)
Lower secondary school	15 (1.66)
Other	17 (1.88)
Marital status	
Single	331 (36.62)
Married	334 (36.95)
In relationship	181 (20.02)
Separated/divorced	43 (4.76)
Widowed	7 (0.77)
Other	8 (0.89)
Occupational status	
Employed	622 (68.81)
Unemployed	25 (2.77)
Retired	66 (7.3)
In education	191 (21.13)
Mental health disorder	
No	700 (77.43)
Yes*	204 (22.57)
Affective (e.g. depression, dysthymia, bipolar)	125 (46.47)
Anxiety (e.g. anxiety, phobia, panic) and OCD	45 (16.73)
Trauma-/stress-related (PTBS, adjustment, burnout)	42 (15.61)
Neurodevelopmental (e.g. AD(H)S, autism)	22 (8.18)
Eating (e.g. anorexia, binge)	15 (5.58)
Personality (e.g. borderline, avoidant)	6 (2.23)
Somatoform (e.g. somatoform, pain, hypochondria)	5 (1.86)
Others	9 (3.35)
Personal positioning with regard to climate change	
Strongly as climate change denier	5 (0.55)
More as climate change denier	6 (0.66)
Neutral/Neither	248 (27.43)
More as climate change activist	490 (54.2)
Strongly as climate change activist	155 (17.15)
Information time	
0–30 min.	373 (41.26)
30–60 min.	238 (26.33)
1–2 h	120 (13.27)
2–4 h	91 (10.07)
4–7 h	46 (5.09)
> 7 h	36 (3.98)
GAD-7	
No (0–4)	379 (41.93)
Mild (5–9)	319 (35.29)
Moderate (10–14)	137 (15.16)
Severe (15–21)	69 (7.63)
PHQ-9	
No (0–4)	413 (45.69)
Mild (5–9)	296 (32.74)
Moderate (10–14)	131 (14.49)
Moderately severe (15–19)	45 (4.98)
Severe (20–24)	19 (2.10)

⁎ Note. As some participants listed multiple diagnoses, the total number of diagnoses exceeds the number of positive answers on having a mental disorder.

### Regression analyses

Sample size for the regression models was *N* = 885 as the inter/diverse gender category (*n* = 19) was previously excluded for statistical reasons. The regression model with CC-MMDS as dependent variable reached an adjusted *R*² of 0.609, *F*(18,866) = 77.44, *p* < .001, Cohen’s *f* = 1.25. The regression model with DT as dependent variable reached an adjusted *R*² of 0.337, *F*(18,866) = 26.00, *p* < .001, Cohen’s *f* = 0.71. The regression model with GAD-7 as dependent variable reached an adjusted *R*² of 0.485, *F*(18,866) = 47.16, *p* < .001, Cohen’s *f* = 0.97. The regression model with PHQ-8 as dependent variable reached an adjusted *R*² of 0.500, *F*(18,866) = 50.13, *p* < .001, Cohen’s *f* = 1.00. The effect sizes of all models correspond to a strong effect size according to Cohen (1992). The standardized regression coefficients and Bonferroni-Holm adjusted *p*-values based on robust standard errors of the four regression models are presented in Table 2. Additional regression results and confidence intervals with both subscales of the CC-MMDS are presented in the supplementary material.

**Table 2:** Standardized regression coefficients and p-values based on robust standard errors (*N* = 885).

Variables	CC Distress		General Distress					
	CC-MMDS		DT		GAD-7		PHQ-8	
	Std. β	*p*	Std. β	*p*	Std. β	*p*	Std. β	*p*
(Intercept)	−0.059	< 0.001	−0.062	< 0.001	−0.158	< 0.001	−0.079	< 0.001
Age	.046	1.00	−0.009	1.00	−0.029	1.00	−0.014	1.00
Gender (ref: male)	.085	1.00	.090	1.00	.227	< 0.01	.114	1.00
Trust in government	−0.212	< 0.001	−0.092	.999	−0.032	1.00	−0.018	1.00
Subjective level of information	.003	1.00	.079	.999	−0.002	1.00	.019	1.00
CERQ-A	.064	.284	−0.066	1.00	−0.006	1.00	.019	1.00
CERQ-BO	.058	1.00	−0.010	1.00	.014	1.00	.010	1.00
CERQ-C	.322	< 0.001	.020	1.00	.111	.262	.103	.471
CERQ-PRa	−0.040	1.00	.039	1.00	.038	1.00	.082	.368
CERQ-PRf	−0.035	1.00	−0.018	1.00	.011	1.00	.026	1.00
CERQ-Ru	.156	< 0.001	.068	1.00	.112	.249	.039	1.00
CERQ-PP	−0.154	< 0.001	.034	1.00	.044	1.00	.045	1.00
CERQ-RP	.123	< 0.01	.040	1.00	−0.006	1.00	−0.918	1.00
CERQ-SB	.094	< 0.05	−0.012	1.00	.002	1.00	.019	1.00
SOC-L9	−0.060	1.00	−0.486	< 0.001	−0.528	< 0.001	−0.539	< 0.001
LOC-E	−0.085	.244	−0.003	1.00	−0.001	1.00	−0.019	1.00
LOC-I	.008	1.00	−0.100	.421	−0.114	.062	−0.030	1.00
RS-11	.062	1.00	−0.072	1.00	−0.042	1.00	−0.206	< 0.05
GSE	−0.001	1.00	−0.062	1.00	−0.148	.053	.012	1.00

Note. *p*-values are Bonferroni-Holm corrected. CC-MMDS, climate change – man-made disaster-related distress scale; DT, distress thermometer; GAD-7, generalized anxiety disorder scale; PHQ-8, patient health questionnaire; CERQ-, cognitive emotion regulation questionnaire-(acceptance, blaming others, catastrophizing, positive reappraisal, positive refocusing, rumination, putting into perspective, refocusing on planning, self-blame); SOC-L9, sense of coherence scale – short form; LOC-, locus of control-(external, internal); RS-11, resilience scale; GSE, general self-efficacy scale.

#### Diverging correlates

To discriminate between CC-MMDS and the different aspects of general distress (assessed with DT, GAD-7, PHQ-8), different correlates are of main interest. Some factors were significantly correlated only with CC-MMDS. These were trust in government and the emotion regulation strategies catastrophizing, rumination, putting into perspective, refocusing on planning, and self-blame. While catastrophizing, rumination, refocusing on planning, and self-blame were positively correlated, trust in government and putting into perspective were negatively correlated.

There were also factors that were significantly correlated with general distress measures, but not with CC-MMDS. Female gender, for example, was only significantly positively correlated with GAD-7. SOC showed a significant negative association with all outcome variables except CC-MMDS. The RS-11 showed a significant negative association with PHQ-8, only.

Age, subjective level of information, the emotion regulation strategies acceptance, blaming others, positive reappraisal, and positive refocusing, external and internal LOC, and GSE were not significantly correlated with any of the dependent variables.

## Discussion

Mental health problems due to climate change, like CC distress, gain more and more relevance in research and health care. However, clarity about the specific correlates of these new phenomena and about their distinction to general distress is still lacking. The goal of the present study was, therefore, to provide more insight into CC distress, compare it with general distress, and to investigate possible associated factors.

Several factors were significantly associated with CC distress, only. Hereby, it is important to differentiate between positively and negatively associated factors. Factors that were in a positive relation with CC distress were the emotion regulation strategies catastrophizing, rumination, refocusing on planning, and self-blame. While catastrophizing correlated with both subscales of the CC-MMDS, rumination correlated specifically with the psychological distress subscale. The positive correlations align with previous research indicating that such neurotic strategies as circulating thoughts usually play a role in anxiety and mood disorders (Garnefski & Kraaij, 2006b; Olatunji et al., 2013; Rodas et al., 2022). Additionally, they are in line with a previous study finding a significant positive correlation of the CC-MMDS with neuroticism (Beckord et al., 2024). However, despite these established associations, corresponding correlations with generalized anxiety or depression were missing in the present study. One possible methodological explanation for this discrepancy may be the framing of the CERQ items in the context of climate change. This context may have lead to amplified correlations with CC distress rather than general distress measures. Thus, factors like the situational context seem to play a specifically relevant role in assessing emotion regulation and its associations with psychological outcomes. Nevertheless, neuroticism and related cognitive-emotional strategies seem to be vulnerability factors linked to heightened CC distress, thus, representing promising targets for psychotherapy.

The positive association with refocusing on planning may result from a greater awareness of climate change and, thus, may be related to feelings of distress and despair, which could explain the positive relationship. As an emotion regulation strategy, it may also reflect an attempt to cope with CC distress, which, however, only becomes adaptive in a second step of action taking. This would be when thinking about taking action actually leads to action, which in turn would boost feelings of self-efficacy. Until then, the mere deliberation may not be enough to serve as a protective mechanism against CC distress. It is important to note that refocusing on planning was only correlated with the subscale change of existing belief systems but not with the subscale psychological distress of the CC-MMDS. This may indicate that refocusing on planning involves changes in belief systems, possibly through directing attention towards action- or solution-oriented thinking, while at the same time shifting attention away from psychological distress rather than directly reducing it. The direction of their relationship and influence should be explored further in future research, for example through longitudinal designs.

Responsibility in the sense of self-blame seems to play a specifically important role for CC distress (Homburg et al., 2007), especially in the context of moral distress – the feeling that one is powerless to act according to one’s moral values (Labarthe & Marks, 2024). Notably, self-blame leads to increased levels of CC-specific psychological distress but not to a change in existing belief systems or increased levels of general distress. This supports the idea that moral distress in the context of climate change is a key factor driving self-blame, possibly through amplifying feelings of shame, powerlessness or inadequacy (Banwell & Eggert, 2024). This emotional response can in turn lead to increased levels of psychological distress in relation to climate change, while at the same time leaving existing core beliefs intact.

Factors that were negatively associated with CC distress were distancing strategies like trust in government and putting into perspective, while trust in government can also be considered within the framework of responsibility. Previous research showed that denial or shift of guilt, putting into perspective, and trusting that (powerful) others are in control induce distancing effects which can mitigate distress and function as coping strategies (Bäuerle et al., 2020; Homburg et al., 2007; Martin & Dahlen, 2005). This would also match the finding that increased self-blame is associated with increased CC distress. Trusting in others would, next to holding them responsible, imply that one trusts them to handle climate change well, thereby being associated with lower levels of CC distress. However, those distancing strategies should be viewed as reactive or self-protective mechanisms rather than successful coping strategies (Gebhardt et al., 2023; Klöckner et al., 2010; Wullenkord & Reese, 2021).

Finally, some factors were only correlated with measures of general distress, but not with CC distress. These were gender, SOC, and resilience. Regarding gender, women reported higher general anxiety levels than men, being in line with previous research showing elevated levels of anxiety in women in general and during crisis (Löwe et al., 2008; Schweda et al., 2021). However, women did not report higher levels of CC distress, general distress, or depression. This suggests that, while women may be more susceptible to general anxiety, this risk effect appears to be limited to anxiety and does not extend to other forms of distress, including distress specifically related to climate change. This supports the idea that general anxiety and CC distress are distinct constructs, influenced by different underlying mechanisms and associated factors.

SOC is another diverging correlate; while it was not associated with CC distress, it was associated with reduced general distress, anxiety, and depression symptoms. Usually, SOC is a protective factor from distressing emotions or in distressing events as it helps to cope with stress and mobilize resources (Schumacher et al., 2000). However, the diverging correlation may be attributed to the context of assessment. While factors like SOC are usually addressed from an individualistic point of view, in the context of climate change, it may be more appropriate to view them from a relational perspective (Kałwak & Weihgold, 2022). Thus, SOC as an individualistic trait may become irrelevant in a relational global context such as climate change.

The same explanation may apply to the missing association of resilience with CC distress in contrast to the negative association with general depression symptoms: While individuals may consider themselves resilient in personal situations, climate change is a global problem requiring collective action – thereby rendering such individualistic traits seemingly irrelevant or insufficient. A related methodological explanation is that SOC, LOC, resilience, and GSE were framed in a general way, whereas CERQ was framed in the context of climate change. This differential framing may have amplified associations between the CERQ and the CC-MMDS, thereby potentially overshadowing existing weaker associations with other measures. This interpretation of potentially overshadowed associations is supported by findings from the previous validation study (Beckord et al., 2024), which showed a significant negative correlation between the SOC and the CC-MMDS. Thus, when measured more exclusively, general constructs such as SOC may indeed relate meaningfully with CC distress and should be investigated further in future research.

Unexpectedly, age, subjective level of information about climate change, acceptance, blaming others, positive reappraisal, positive refocusing, LOC, and GSE were not correlated with any of the outcomes. The lack of association between CC distress and age is contradictive to previous research indicating that especially younger people aged under 25 suffer from an increased distress due to climate change (Hickman et al., 2021; Koder et al., 2023). This may be due to age being measured continuously and the model testing a linear relationship. The association between CC distress and age may, however, not be linear. In addition to younger people, older adults are frequently presented as a vulnerable group particularly susceptible to climate-related physical and mental health consequences due to socio-economical and physical vulnerabilities (Clayton et al., 2021). A closer investigation of the relationship between CC distress and age using a scatter plot revealed, however, no apparent non-linear relationship, nor any relationship whatsoever (see supplementary material, Figure 1). Thus, factors other than age seem to be more important for the experience of CC distress. Despite their objective vulnerability, older people subjectively may not perceive themselves as affected (Clayton et al., 2021), explaining the absence of a correlation. Additionally, older and younger people seem to experience different consequences, making it difficult to find a clear and linear relationship. While younger people seem to be more distressed by climate change, older people seem more functionally impaired (König et al., 2024). A more thorough comparison between distinct age groups might reveal clearer differences and provide more insight.

Subjective level of information about climate change was neither correlated with general distress measures nor with CC distress, despite being posed in the context of climate change. Thus, the level of information about climate change seems not to influence the level of experienced distress. However, the scale more specifically covered knowledge of measures to mitigate climate change rather than knowledge of, for example, the seriousness or consequences of climate change. It is possible that the type of information about climate change influences how it is correlated with distress. This should be investigated in more detail in future research.

The emotion regulation strategies acceptance, blaming others, positive reappraisal, and positive refocusing were also neither correlated with CC distress nor with general distress, anxiety, or depression. The missing association with acceptance is in contrast to previous research indicating that acceptance usually is a coping response, which mitigates distressing thoughts or emotions (Garnefski et al., 2001). In the context of climate change, however, researchers suggest that the items of the subscale acceptance (e.g. “I think that I must learn to live with it”) may also reflect a certain degree of resignation and help- and hopelessness (Clayton et al., 2017; Martin & Dahlen, 2005). Additionally, acceptance of climate change may also imply a stronger awareness of climate change, which would also lead to increased levels of distress. Thus, a contradicting association could have been expected. However, it is possible that the association is weak and has been masked or suppressed in the present model. Future studies with a more focused design should aim to clarify this. Blaming others would also have been expected to correlate with CC distress, given the relationship of self-blame and trust in government with CC distress. Additionally, previous research showed a positive relationship between blaming others and distress during crisis (Rus et al., 2020). Positive reappraisal showed no significant correlations in this study even though it was associated with depression symptoms in previous research (Garnefski & Kraaij, 2007; Troy et al., 2010). It is possible that, while depressed individuals may engage more frequently in reappraisal, it is independent from the efficacy of it (Troy et al., 2010). Thus, in the present context it may not be a successful coping strategy and may also depend on individual factors or the level of experienced stress (Garnefski & Kraaij, 2007; Troy et al., 2010). That positive refocusing is also not correlated with either of the outcomes is consistent with previous research showing no significant relationship with depression or anxiety (Garnefski & Kraaij, 2007). Future research should explore the complex relationship between emotion regulation strategies, general and specific distress, and high and low distress further.

The absence of significant associations of the factors LOC and GSE with CC distress may as well be attributed to the context of assessment, rendering those traits irrelevant in a global context such as climate change. Additionally, in the context of climate change, general self-efficacy may be less important than the specific concepts of environmental self-efficacy (Clayton et al., 2017; Sawitri et al., 2015) or collective efficacy (Clayton et al., 2021). Regarding LOC, the present results also contradict previous research showing internal control to protect from general anxiety and distress (Iles-Caven et al., 2023; Krampe et al., 2021). It is possible that these protective effects were too small to be detected in the present study sample and model as the study sample was not distressed enough. It would be interesting for further research to compare a highly distressed and low distressed group.

There are some limitations that should be noted. For once, this study’s design is a cross-sectional one, thereby limiting the possibility to draw causal conclusions. Definitive interpretations of directions remain tentative and in need of a longitudinal design. Another possible limitation is the focus on a German-speaking and indirectly affected sample. As contextual factors, such as national climate policies or recent environmental disasters, can shape how CC distress is experienced, this may have influenced the results and limits the generalizability to other cultural or regional contexts. Nevertheless, indirect psychological consequences can also lead to significant distress and are increasingly recognized and important to understand. However, future research should also explore cross-regional differences in CC distress. One way to facilitate comparisons of directly and indirectly affected regions would be to validate the CC-MMDS in additional languages and cultural contexts. Next to this, there may be a possible selection bias, as the sample is unbalanced towards an overrepresentation of women and highly educated persons, thereby limiting the generalizability of results. It would be interesting to investigate how men and women differ in the usage of emotion regulation strategies to regulate CC distress. Additionally, only persons interested in the topic of climate change would take part, leading to imbalances in the population such as the observed overrepresentation of “climate change activists”. Furthermore, the terms “climate change activist” and “climate change denier” were not explicitly defined in the questionnaire, which could have resulted in participants interpreting these labels differently based on personal, cultural, or political perspectives. This variability in interpretation should be taken into account when evaluating the results. It may also apply that a social desirability bias has distorted responses. Future research should try to generalize the results through recruiting a more representative sample. Another important limitation is that the scales trust in government, subjective level of information, and CERQ were posed in the context of climate change, while the other questionnaires were not. Thus, this may have biased participants’ responses and inflated associations while potentially overshadowing existing weaker associations with other variables. Consequently, the observed correlations may be partly attributed to the contextual framing and should be interpreted with caution. Nevertheless, the correlation between CC distress and CERQ reveals valuable insights into the relevant (dys-)functional regulation strategies for CC distress, making them promising targets for psychotherapy. Future studies with larger samples and consistently framed questionnaires are needed to minimize context effects and allow for a more accurate investigation of these relationships. Finally, even though specific associated factors differ between CC distress and general distress, the experience of both may cumulate to a stronger and broader experience of distress. This probable interplay was not adequately captured in the present study. Future research could investigate the existence of latent sub-groups, individuals with pre-existing mental health conditions, and compare high and low CC distress groups for further clarification.

Despite of these limitations, the present study provides an important insight into the correlates of CC distress and serves as a valuable contribution towards a conceptual clarification and delineation of CC distress. The results of the present study support that CC distress and general distress are overlapping but distinct constructs as indicated by previous research (Beckord et al., 2024; Contreras et al., 2024). As CC distress is a complex construct comprising a lot of different emotions (Beckord et al., 2024; Koder et al., 2023), we propose to continually use CC distress as umbrella term for negative cognitive and emotional reactions to climate change. Thus, eco-anxiety would be falling under the scope of CC distress, more specifically relating to feelings of environmental doom as proposed by the American Psychological Association (Clayton et al., 2017). As psychotherapists will experience a rise in clients expressing CC distress (Koder et al., 2023), while at the same time reporting that they feel inadequately prepared to address these issues (Trost et al., 2024), this research will aid psychotherapists by shedding light on possible resources or targets for psychotherapy. Specifically, the identified emotion regulation strategies, such as catastrophizing, rumination, and self-blame, offer promising targets for tailored interventions. For example, interventions that reduce maladaptive rumination or promote adaptive coping could reduce CC distress. While there are first promising trials for therapy for CC distress, e.g. tailored Internet-delivered cognitive-behavioural therapy (Lindhe et al., 2023), an improved conceptual clarity can further advance tailored treatment. Moreover, these insights may also aid in the development of specialized training programs for psychotherapists in climate-conscious psychotherapy. Beyond clinical contexts, understanding factors like trust in government can inform public policy and communication efforts. For instance, this insight can be used to design transparent communications that build trust among the public. Altogether, these implications highlight the importance of interdisciplinary approaches that integrate psychological support, professional trainings, and informed policy to address the growing mental health impact of climate change.

## Conclusion

The present study provides important clarity on the specific correlates of CC distress. CC distress is influenced by a variety of factors that are distinct from those of general distress. Consequently, a clear distinction between CC distress and general distress can be established. More specifically, trust in government and emotion regulation strategies seem to significantly influence the level of experienced CC distress, while SOC seems to be especially relevant for general distress. Those associated factors can be promising targets for intervention strategies and psychotherapeutic contexts. Moreover, they demonstrate how important it is for economic and political sectors, e.g. in government, industry, and health care, to take responsibility and to communicate effectively. Finally, we propose to continually use CC distress as umbrella term for negative cognitive and emotional reactions to climate change.

## Abbreviations

CC distress = climate change distress

CC-MMDS = Climate Change – Man-Made Disaster-Related Distress Scale

CC-MMDS-PD = CC-MMDS-Psychological Distress subscale

CC-MMDS-CBS = CC-MMDS-Change of existing Belief Systems subscale

DT = Distress Thermometer

GAD-7 = Generalized Anxiety 7-item Scale

PHQ-8 = Patient Health Questionnaire-8

CERQ = Cognitive Emotion Regulation Questionnaire

SOC(-L9) = Sense of Coherence (Scale Short Form)

LOC = Locus of Control Scale

RS = Resilience Scale

GSE = General Self-Efficacy Scale

## Funding

This work was supported by the Drs. Graute and Graute-Oppermann-Foundation [grant number T0277 – 44.332].

## Data statement

The data supporting the presented results is available from the CA on reasonable request.

## Ethical standards

This study is approved by the Ethical Committee of the Medical Faculty of the University of Duisburg-Essen (23–11,342-BO).

## CRediT authorship contribution statement

Conceptualization: JB, MT & AB. Data curation: JB. Formal analysis: JB. Funding acquisition: JB, EMS, & AB. Investigation: JB, JBK, EMS, MT & AB. Methodology: JB, NG, CN, JBK, EMS, MT & AB. Project administration: JB, EMS, MT & AB. Supervision: EMS, MT & AB. Validation: JB, EMS, MT & AB. Visualization: JB. Writing - original draft: JB. Writing - review & editing: JB, NG, CN, JBK, EMS, MT & AB.

## Declaration of competing interest

The authors declare that they have no known competing financial interests or personal relationships that could have appeared to influence the work reported in this paper.

## Appendix Supplementary materials

Image, application 1

Image, application 2

## Footnotes

1 r_SB_ = spearman-brown-coefficient
