# Supplementary material for: Correlates of climate change distress: The difference to general distress
Source: Int J Clin Health Psychol. 2025 Aug 5;25(3):100613. doi: 10.1016/j.ijchp.2025.100613 (PMC12351118; doi:10.1016/j.ijchp.2025.100613)
Supplement: Supplementary file 2 [file mmc2.docx]

**Table 1.** Standardized regression coefficients and confidence intervals based on robust standard errors (*N* = 885).

| Variables | CC-MMDS | | CC-MMDS-PD | | CC-MMDS-CBS | | DT | | GAD-7 | | PHQ-8 | |
| --- | --- | --- | --- | --- | --- | --- | --- | --- | --- | --- | --- | --- |
| Adjusted *R*² | .6088 | | .5821 | | .4994 | | .3373 | | .4845 | | .5001 | |
|  | Std. β | 95% KI | Std. β | 95% KI | Std. β | 95% KI | Std. β | 95% KI | Std. β | 95% KI | Std. β | 95% KI |
| (Intercept) | -.059*** | [-.14; .02] | -.094*** | [-.18; -.01] | .026*** | [-.06; .12] | -.062*** | [-.17; .04] | -.158*** | [-.25; -.07] | -.079*** | [-.17; .01] |
| Age | .046 | [.00; .09] | .042 | [-.01; .09] | .046 | [-.01; .10] | -.009 | [-.07; .05] | -.029 | [-.08; .02] | -.014 | [-.07; .04] |
| Gender (ref: male) | .085 | [-.01; .18] | .135 | [.03; .23] | -.037 | [-.15; .07] | .090 | [-.04; .22] | .227** | [.12; .34] | .114 | [.00; .22] |
| Trust in government | -.212*** | [-.26; -.16] | -.151*** | [-.20; -.10] | -.307*** | [-.37; -.25] | -.092 | [-.16; -.02] | -.032 | [-.09; .03] | -.018 | [-.08; .04] |
| Subj. level of information | .003 | [-.04; .05] | -.029 | [-.08; .02] | .073 | [.02; .13] | .079 | [.02; .14] | -.002 | [-.05; .05] | .019 | [-.03; .07] |
| CERQ-Acceptance | .064 | [.02; .11] | .069 | [.02; .11] | .040 | [-.01; .09] | -.066 | [-.12; -.01] | -.006 | [-.06; .04] | .019 | [-.03; .07] |
| CERQ-Blaming others | .058 | [.01; .10] | .045 | [.00; .09] | .075 | [.02; .13] | -.010 | [-.07; .05] | .014 | [-.04; .07] | .010 | [-.04; .06] |
| CERQ-Catastrophizing | .322*** | [.26; .38] | .337*** | [.27; .40] | .231*** | [.16; .30] | .020 | [-.06; .10] | .111 | [.04; .18] | .103 | [.03; .17] |
| CERQ-Positive reappraisal | -.040 | [-.09; .01] | -.042 | [-.09; .01] | -.030 | [-.09; .03] | .039 | [-.03; .10] | .038 | [-.02; .10] | .082 | [.03; .14] |
| CERQ-Positive refocusing | -.035 | [-.08; .01] | -.050 | [-.10; .00] | .004 | [-.05; .06] | -.018 | [-.08; .04] | .011 | [-.04; .06] | .026 | [-.03; .08] |
| CERQ-Rumination | .156*** | [.10; .21] | .190*** | [.13; .25] | .057 | [-.01; .12] | .068 | [-.01; .14] | .112 | [.04; .18] | .039 | [-.03; .10] |
| CERQ-Putting into perspective | -.154*** | [-.21; -.10] | -.167*** | [-.22; -.11] | -.098 | [-.16; -.04] | .034 | [-.03; .10] | .044 | [-.02; .10] | .045 | [-.01; .10] |
| CERQ-Refocusing on planning | .123** | [.06; .18] | .079 | [.02; .14] | .195*** | [.13; .26] | .040 | [-.04; .12] | -.006 | [-.08; .06] | -.918 | [-.09; .05] |
| CERQ-Self-blame | .094* | [.05; .14] | .105** | [.06; .15] | .055 | [.00; .11] | -.012 | [-.07; .05] | .002 | [-.05; .05] | .019 | [-.03; .07] |
| Sense of coherence scale | -.060 | [-.12; .00] | -.054 | [-.12; .01] | -.064 | [-.13; .00] | -.486*** | [-.56; -.41] | -.528*** | [-.60; -.46] | -.539*** | [-.61; -.47] |
| External locus of control | -.085 | [-.13; -.04] | -.076 | [-.13; -.03] | -.087 | [-.14; -.03] | -.003 | [-.07; .06] | -.001 | [-.06; .06] | -.019 | [-.07; .04] |
| Internal locus of control | .008 | [-.04; .06] | .007 | [-.05; .06] | .010 | [-.05; .07] | -.100 | [-.17; -.03] | -.114 | [-.17; -.05] | -.030 | [-.09; .03] |
| Resilience scale | .062 | [.00; .13] | .068 | [.00; .13] | .038 | [-.04; .11] | -.072 | [-.16; .01] | -.042 | [-.12; .03] | -.206* | [-.28; -.13] |
| General self-efficacy scale | -.001 | [-.07; .06] | -.021 | [-.09; .05] | .044 | [-.03; .12] | -.062 | [-.15; .02] | -.148 | [-.22; -.07] | .012 | [-.06; .09] |

Note. * *p* < .05, ** *p* < .01, *** *p* < .001. *p*-values are Bonferroni-Holm adjusted. CC-MMDS-, climate change – man-made disaster-related distress scale – (psychological distress, change of existing belief systems); DT, distress thermometer; GAD-7, generalized anxiety disorder scale; PHQ-8, patient health questionnaire; CERQ, cognitive emotion regulation questionnaire.

**Figure 1.** Relationship between CC distress and age (N = 885).
